# Construction and comparative evaluation of different activity detection methods in brain FDG-PET

**DOI:** 10.1186/s12938-015-0073-x

**Published:** 2015-08-18

**Authors:** Hans-Georg Buchholz, Fabian Wenzel, Martin Gartenschläger, Frank Thiele, Stewart Young, Stefan Reuss, Mathias Schreckenberger

**Affiliations:** Department of Nuclear Medicine, University Medical Center Mainz, Langenbeckstrasse 1, 55101 Mainz, Germany; Philips Research Laboratories, Hamburg, Germany; Philips Research Laboratories, Aachen, Germany; Philips Healthcare, Hamburg, Germany

**Keywords:** FDG, PET/CT, Brain mapping, Reference database, Alzheimer’s disease

## Abstract

**Aim:**

We constructed and evaluated reference brain FDG-PET databases for usage by three software programs (Computer-aided diagnosis for dementia (CAD4D), Statistical Parametric Mapping (SPM) and NEUROSTAT), which allow a user-independent detection of dementia-related hypometabolism in patients’ brain FDG-PET.

**Methods:**

Thirty-seven healthy volunteers were scanned in order to construct brain FDG reference databases, which reflect the normal, age-dependent glucose consumption in human brain, using either software. Databases were compared to each other to assess the impact of different stereotactic normalization algorithms used by either software package. In addition, performance of the new reference databases in the detection of altered glucose consumption in the brains of patients was evaluated by calculating statistical maps of regional hypometabolism in FDG-PET of 20 patients with confirmed Alzheimer’s dementia (AD) and of 10 non-AD patients. Extent (hypometabolic volume referred to as cluster size) and magnitude (peak z-score) of detected hypometabolism was statistically analyzed.

**Results:**

Differences between the reference databases built by CAD4D, SPM or NEUROSTAT were observed. Due to the different normalization methods, altered spatial FDG patterns were found. When analyzing patient data with the reference databases created using CAD4D, SPM or NEUROSTAT, similar characteristic clusters of hypometabolism in the same brain regions were found in the AD group with either software. However, larger z-scores were observed with CAD4D and NEUROSTAT than those reported by SPM. Better concordance with CAD4D and NEUROSTAT was achieved using the spatially normalized images of SPM and an independent z-score calculation. The three software packages identified the peak z-scores in the same brain region in 11 of 20 AD cases, and there was concordance between CAD4D and SPM in 16 AD subjects.

**Conclusion:**

The clinical evaluation of brain FDG-PET of 20 AD patients with either CAD4D-, SPM- or NEUROSTAT-generated databases from an identical reference dataset showed similar patterns of hypometabolism in the brain regions known to be involved in AD. The extent of hypometabolism and peak z-score appeared to be influenced by the calculation method used in each software package rather than by different spatial normalization parameters.

## Background

Voxel-wise comparison of FDG-PET brain images is widely used in neuroimaging to assess disease-specific and -associated changes in metabolism. A user-independent tool for voxel-by-voxel statistical analysis is requested to detect hypo- or hyper-metabolism by comparing patients to healthy control subjects. Software packages such as NEUROSTAT (Department of Radiology, University of Washington, Seattle, Washington, USA) or Statistical Parametric Mapping (SPM; Wellcome Trust Centre for Neuroimaging, London, UK; current version: SPM8) are widely used in the analysis of brain FDG-PET images. For example, SPM was chosen to detect and to differentiate types of dementia [1–4], Huntington’s chorea [5], temporal lobe epilepsy [6] and age-related hypometabolisms [7–11]. Studies focusing on the detection of neurodegenerative diseases [12–15] analyzed data by NEUROSTAT and detected regions of hypometabolism in varying patterns, which were related to the patient’s type of dementia. For example, while glucose metabolism was reduced in temporo-parietal association cortex and posterior cingulate cortex in Alzheimer’s disease (AD), it was additionally reduced in the primary visual cortex of patients suffering from dementia with Lewy bodies (DLB) [16].

For the purposes of user-independent inter-subject comparisons, two major points have to be considered. First, the original images are transformed into a standard coordinate system by warping the individual image to a template image in the reference space. Second, a reference brain FDG database of healthy subjects must be used to detect possible alterations in patients’ brain PET. Furthermore, due to scanner-associated differences in resolution and reconstruction (e.g., algorithm, type of scatter and attenuation correction), the patient data and the reference database ideally should be acquired under the same conditions by the same PET scanner. This was the case in our comparative study presented here.

We evaluated three different software packages, i.e., NEUROSTAT, SPM and a software-prototype called CAD4D (Computer-aided diagnosis for dementia) recently developed by Philips. Native PET/CT brain images of healthy controls served to create three alternative reference FDG brain databases by using NEUROSTAT, SPM or CAD4D, respectively. Then, differences between these software-specific reference databases were assessed. Earlier comparative evaluations of stereotactic normalization procedures [17, 18] did not reveal any major differences between SPM and NEUROSTAT. The latter is restricted to the use of the so-called “Talairach space” [19] as a standard coordinate system, whereas SPM and CAD4D, by default, operate in the “MNI space” (Montreal Neurological Institute, McGill University, Montreal, Canada).

Furthermore, we evaluated the FDG brain reference databases using data from AD- and non-AD patients in two ways. Firstly, we compared individual AD patient data by statistical testing using NEUROSTAT, SPM and CAD4D and newly constructed reference databases (as described above). Secondly, we performed two-sample t-tests between the images normalized by either software of the 37 healthy controls building the reference databases (group 1) and of the 20 AD-patients (group 2) in order to assess overall hypometabolism, the extent of which may depend only on different normalization methods.

**Fig. 1:**
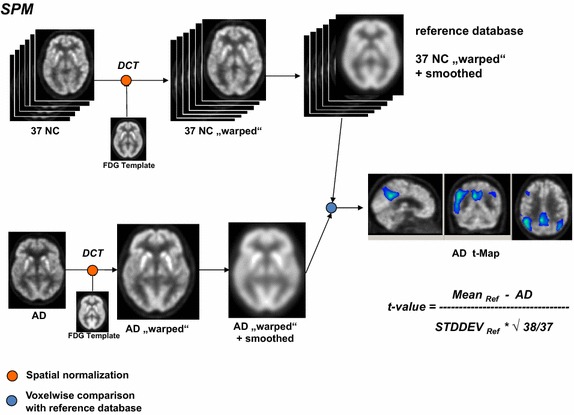
Workflow of SPM software. After spatial normalization, a t-map image was calculated by voxel-by-voxel single subject comparison to reference database

**Fig. 2:**
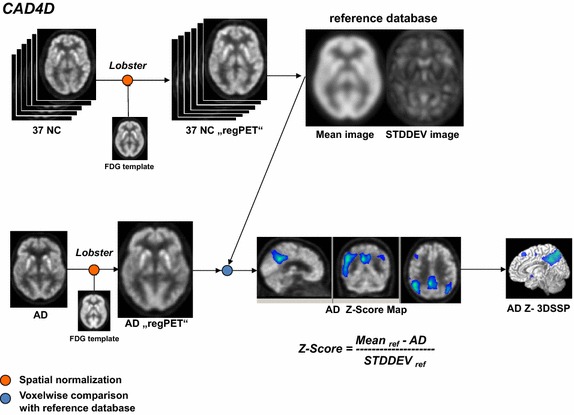
Workflow of CAD4D software. After spatial normalization, the z-score image of an AD patient was calculated by voxel-by-voxel comparison to the reference database created from healthy subjects

**Fig. 3:**
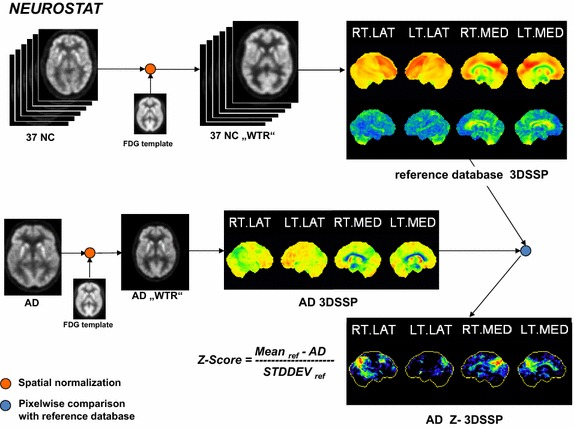
Workflow of NEUROSTAT software. After spatial normalization, z-score 3DSSP was calculated by pixel-by-pixel comparison to reference database 3DSSP

Since SPM does not calculate z-scores directly (see Fig. 1), we also performed a separate calculation of voxel-wise z-scores using the SPM-normalized images for each AD patient and the reference database, and applied the same formula as shown in CAD4D and NEUROSTAT (see Figs. 2, 3).

The aim of the present study therefore was to statistically evaluate patient brain FDG-PET compared to a reference database in an integrated workflow. These methods do not require MRI data which are often not available in the clinical diagnostic routine. Notably, CAD4D, SPM and NEUROSTAT are intended to detect alterations in brain glucose consumption in general, rather than providing a tool for AD diagnosis in particular. In the present study, we used these programs to test the appearance of specific hypometabolism in a group of Alzheimer’s disease patients.

## Methods

### Data acquisition

The data used in this study were collected from three groups of probands or patients, respectively. Group one consisted of 37 healthy controls recruited by advertisements posted in the University Medical Center of the Johannes Gutenberg-University Mainz (Germany) and via newspaper announcements. Interested subjects underwent a psychiatric screening interview. Subjects with a history of psychiatric, neurologic or cognitive disease were excluded, as well as those taking medications that may alter cognitive performance. Structural MRI and neuropsychological tests including trail-making tasks were performed to verify normal ageing status. The mean age was 70 years (range: 50–85 years; 19 females/18 males). All subjects participating in this study provided written informed consent. The study protocol was approved by the radiation protection authorities and by the local ethics committee.

Group two consisted of 20 AD patients (mean age: 64.7 years; range: 50–84 years; 11 females/9 males; Mini Mental State Examination (MMSE): median: 21, range: 3–26) selected by order of appearance from a clinical cohort (Department of Psychiatry, University Medical Center, Mainz, Germany). In the FDG-PET/CT, they showed distinct signs of AD-related hypometabolism (see below).

Group three consisted of ten patients with cognitive impairment (mean age: 67.3 years, range: 51–79 years, 5 female/5 male; MMSE: median: 27 range: 23–29). In the FDG-PET/CT, they showed no distinct signs of AD-related hypometabolism and were therefore classified as non-AD.

We used the standardized protocol for preparation and data acquisition, as described in the European Association of Nuclear Medicine (EANM) procedure guidelines for brain FDG-PET imaging [20]. Thirty minutes after intravenous administration of 150 ± 20 MBq FDG, PET/CT measurements were performed on a Philips Gemini TF16 PET/CT scanner over 15 min. The scanner had an axial field of view of 18 cm and an axial and transversal spatial resolution of 4.3 mm at full width at half maximum (FWHM) [21]. Images were reconstructed with scatter- and CT-based attenuation-correction using the iterative RAMLA 3D algorithm [22], resulting in an image matrix of 128 × 128 × 90 and isotropic voxels of 2 × 2 × 2 mm^3^.

### Creation of normal brain FDG databases

Three software packages with different methods of spatial normalization were used, i.e. CAD4D, SPM and NEUROSTAT.

#### CAD4D

The software of CAD4D for spatial normalization into the MNI space, named “Lobster” (Locally Optimal B-Spline-based Transformations for Elastic Registration), uses a 12-parameters affine transformation followed by a non-linear two-step b-splines-based procedure [23]. Spatially normalized images of the reference datasets were intensity-scaled to the median value using a predefined grey-white matter mask (labeled “regPET” in Fig. 2, [18]). The image dimension was 91 × 109 × 91 (MNI space) with isotropic voxels of 2 × 2 × 2 mm^3^. After smoothing with a Gaussian filter of 10 mm width, the reference database was constructed by calculating a MEAN image and a STDDEV image from the 37 reference datasets.

For comparison with NEUROSTAT, normalization to Talairach space (image matrix: 128 × 128 × 60; isotropic voxels of 2.25 × 2.25 × 2.25 mm^3^) was performed by changing the default settings of CAD4D. “regPET” images were smoothed as described above. MEAN and STDDEV images in Talairach space were calculated and used as reference databases in the Talairach version of CAD4D referred to as CAD4D(TAL). For each FDG image of a single patient, CAD4D performed the analysis in one step resulting in three images in 16-bit integer binary NIfTI-1 data format (Neuroimaging Informatics Technology Initiative). These consisted of the spatially normalized FDG image of the patient, a z-score map of hypometabolism from voxel-wise comparison to newly constructed reference database and the corresponding 3D-standard surface projection (3D-SSP) images of the z-score map (see Fig. 2).

#### SPM

**Fig. 4:**
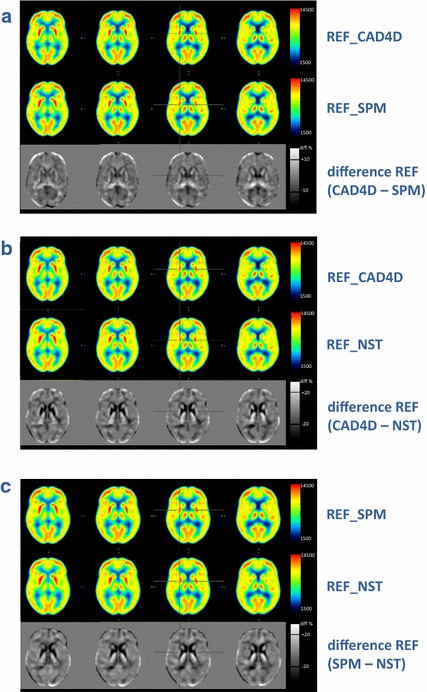
Comparison of 37 reference datasets (REF): **a** normalized with CAD4D or SPM, **b** normalized with CAD4D or NEUROSTAT (NST), **c** normalized with SPM or NEUROSTAT (NST). Difference images depict increased (*white*) or decreased (*black*) metabolic activity

DICOM images obtained from PET/CT workstation were resliced along the virtual line passing the anterior and posterior commissures (AC-PC-line) and converted to 16-bit integer NIFTI format using PMOD3.1 (PMOD Technologies Ltd., Adliswil, Switzerland). Matlab version 7.9 (The Mathworks Inc., MA, USA) and SPM (version SPM8) were used to build the reference database from 37 healthy controls. The spatial normalization by SPM consisted of two steps. First, the optimum of a 12-parameter affine transformation was calculated. Second, non-linear warping to the scanner-specific FDG template was performed using linear combination of three-dimensional discrete cosine transformation (DCT) basis functions yielding deformation fields. Further details were described by Friston and Ashburner [24, 25]. We used the default settings of spatial normalization into MNI space for SPM (number of nonlinear basis functions: 7 × 9 × 7; number of iterations: 16; bounding box: 90 −91, −126 91, −72 109; regularization: medium; voxel sizes: 2 × 2 × 2 mm^3^; image size: 91 × 109 × 91; origin: 46/64/37). Finally, we smoothed the warped images with a Gaussian filter of 10 mm. T-map calculations were performed on voxelwise basis as single subject analysis of an AD patient dataset compared to the reference database [consisting of images of 37 normal healthy controls (see Fig. 1)]. A Matlab program was written to calculate a MEAN image and an STDDEV image for direct comparison to CAD4D (see Fig. 4). Intensity scaling was applied to the median value, which we obtained using the SPM extension toolkit, Marsbar (http://www.marsbar.sourceforge.net/), and the grey-white-matter mask from CAD4D. In addition, we calculated z-score images using SPM’s Imcalc function and the formula used in CAD4D and NEUROSTAT. For the comparison with NEUROSTAT, we modified the default settings to enable SPM to spatially normalize images into the Talairach space referred to as SPM(TAL), as proposed by Ishii et al. [26]. In addition, we applied the procedures used to establish the SPM reference database (smoothing, scaling to median value) and to calculate z-score images as described above.

#### NEUROSTAT

The current graphical user interface (GUI) version of NEUROSTAT (iSSP 3.5), including version 7.0 of spatial normalization software “stereo.exe” was chosen for comparison to SPM and CAD4D. Spatial normalization into the Talairach space consisted of a linear scaling and nonlinear warping. First, the location of the midsagittal plane was determined and the AC-PC-line was iteratively estimated. Since this algorithm was developed when the field-of-views of PET scanners typically did not cover the whole brain, the dorso-ventral extent of the brain was estimated by contour matching and linear scaling. In the second step, nonlinear warping was performed by using predefined stretching centers in white matter and gray matter surface landmarks [27–29]. The resulting spatially normalized image had a matrix of 128 × 128 × 60 isotropic voxels with size of 2.25 × 2.25 × 2.25 mm^3^. Eight 3D stereotactic surface projections (3D-SSPs) were calculated. A normal database was created using GUI tool DBbuilder and 3D-SSPs from the 37 NC. Intra-set intensity scaling was performed either to global brain (GLB), thalamus, cerebellum, pons or sensorimotor cortex, thus resulting in five different reference databases. Figure 3 shows the workflow for NEUROSTAT. For illustrative purposes, only the reference database scaled to GLB and only four out of eight 3D-SSPs are displayed. Z-score calculation in NEUROSTAT was performed on a pixel-wise basis by comparing 3D-SSPs of a single subject to the reference database 3D-SSPs [29].

For comparison to CAD4D and SPM, we used the reference database 3D-SSPs with intra-set intensity scaling to GLB. In addition, the warped images (WTR) were saved for volumetric spatial normalization comparisons with the reference databases created by CAD4D and by SPM.

### FDG template

Spatial normalization in CAD4D and SPM was performed using the same scanner-specific FDG template image. This symmetrical template was previously created by our group using CAD4D by normalizing images of 20 young healthy controls (age <40 years), as previously described for a healthy control group [30]. Additionally for the use with the NEUROSTAT software, the new FDG template was also transformed into the Talairach space using the spatial normalization procedure of SPM8 (see SPM section) and NEUROSTAT’s standard template.

### Comparisons of reference databases

Spatially normalized images of the 37 reference datasets in both, MNI and Talairach space were scaled to their median value. Paired t-tests were performed with SPM8 (p < 0.001, FWE-corrected) in order to assess systematic differences in the FDG-distribution in brain caused by the different spatial normalization procedures implemented in CAD4D, SPM and NEUROSTAT.

### Statistical testing of patient data

Stereotactic normalizations into MNI and Talairach space within each software package were performed as described above. First, patient image data were analyzed by CAD4D, SPM and NEUROSTAT using the reference databases constructed previously. CAD4D and NEUROSTAT calculate z-score directly, whereas SPM uses the “two sample *t* test” model by calculating voxel-wise t-maps from the single-subject-analysis (group 1: 37 healthy controls, “group 2”: one of the 20 AD or 10 non-AD) and using SPM’s function “spm_t2z.m” to transform t- into z-scores.

The resulting statistical maps were evaluated by extracting the maximum z-score and the total cluster size of hypometabolism at a threshold of z > 3 using PMOD software and an iso-intensity volume of interest (VOI).

In addition, we conducted comparisons between the healthy controls group and AD patients group (AD20) using the normalized FDG images of CAD4D, SPM or NEUROSTAT in order to assess possible differences based only on different normalization procedures.

Differences in statistical methods (direct z-score calculation used in CAD4D and NEUROSTAT versus t-statistics and t–to-z transformation used in SPM) result in diversities in the detected AD-related hypometabolism (see “Results”). Therefore, we produced SPM z-score images directly, using the formula in Fig. 2, referred to as “SPM calc” (see Table 1).

**Table 1:** Evaluation of each of 20 AD subjects

MNI	CAD4D		SPM		SPM calc	
#AD	Max z-score	Cluster size (voxels)	Max t-score (z-score)	Cluster size (voxels)	Max z-score	Cluster size (voxels)
1	7.23	4360	6.25 (5.11)	3198	6.36	4682
2	7.90	20,991	8.92 (6.44)	20,839	9.04	24,226
3	5.57	2495	6.74 (5.39)	2485	6.86	3340
4	5.80	6675	6.60 (5.31)	4556	6.71	6203
5	7.48	6251	6.76 (5.39)	4796	6.87	6628
6	7.53	17,525	7.55 (5.81)	15,977	7.60	19,848
7	6.03	3038	5.94 (4.93)	2257	6.04	3103
8	7.90	25,495	11.30 (7.30)	26,715	11.33	30,529
9	6.62	5260	7.16 (5.61)	5984	7.29	7493
10	7.90	17,031	9.89 (6.83)	15,673	10.27	18,474
11	7.90	20,014	10.59 (7.09)	22,193	10.70	25,979
12	7.90	16,370	9.82 (6.81)	20,141	9.89	23,710
13	7.90	8739	6.60 (5.31)	5990	6.66	8233
14	6.21	2131	6.02 (4.97)	1770	6.10	2895
15	7.63	12,155	7.82 (5.94)	9359	7.91	12,643
16	7.90	15,197	9.46 (6.66)	12,125	9.56	14,529
17	7.90	14,100	8.90 (6.40)	13,161	9.03	15,670
18	7.90	9441	9.16 (6.54)	9707	9.26	9929
19	7.90	2442	8.79 (6.38)	1334	8.86	1764
20	6.68	4117	7.22 (5.64)	2340	7.34	3592
Min		2131		1334		1764
Max		25,495		26,715		30,529
Median		9090		7654		9081
Wilcox			CAD4D/SPM	0.057	CAD4D/SPMc	0.012*
					SPM/SPMc	<0.001*

TAL	CAD4D		SPM		SPM calc		NEUROSTAT
#AD	Max z-score	Cluster size (voxels)	Max t-score (z-score)	Cluster size (voxels)	Max z-score	Cluster size (voxels)	Max z-score
1	6.88	1861	5.56 (4.69)	1179	6.23	2989	5.07
2	7.90	10,106	8.36 (6.19)	11,097	8.96	14,565	8.15
3	6.36	1746	4.92 (4.27)	681	6.58	1805	5.69
4	6.09	1933	5.14 (4.42)	1280	7.20	3441	5.63
5	6.71	3078	6.95 (5.50)	1896	6.94	3954	7.85
6	7.67	7757	6.94 (5.50)	6299	7.79	12,119	7.48
7	6.38	1033	5.19 (4.46)	699	6.15	1751	5.64
8	7.90	13,275	10.41 (7.03)	15,746	10.85	19,129	7.17
9	6.25	2507	7.02 (5.54)	2279	7.30	4304	5.31
10	7.90	7687	9.00 (6.47)	7662	9.91	11,112	6.66
11	7.90	10,550	7.36 (5.71)	5433	10.63	14,914	8.75
12	7.90	8775	8.64 (6.32)	8190	9.83	13,702	7.71
13	6.89	2996	6.30 (5.14)	2691	6.97	4766	6.26
14	6.58	1863	5.46 (4.63)	625	5.98	1647	5.66
15	7.42	5084	6.61 (5.32)	3808	7.88	8205	8.16
16	7.86	6135	8.03 (6.04)	5074	9.57	8538	7.47
17	7.90	7635	8.98 (6.46)	6774	9.01	9136	10.88
18	7.52	5096	8.35 (6.19)	4816	8.74	6709	8.31
19	6.37	914	8.06 (6.06)	774	7.93	1000	7.03
20	7.00	1027	4.83 (4.21)	745	5.83	1872	4.81
Min		914		625		1000	
Max		13,275		15,746		19,129	
Median		4081		3250		5738	
Wilcox			CAD4D/SPM	0.006*	CAD4D/SPMc	<0.001*	
					SPM/SPMc	<0.001*	

Extracted maximum z-score/t-score and total hypometabolic cluster size at z > 3 using CAD4D and SPM in MNI space (upper table) and in Talairach space (lower table) compared to the maximum z-score in NEUROSTAT’s 3DSSP; max. z-score and cluster sizes from direct z-score calculation of SPM-normalized images (referred to SPM calc) in MNI and Talairach space. Wilcoxson test statistics (Wilcox) was calculated between the cluster sizes of hypometabolism at z > 3, the asterisk indicates a significance of p < 0.05

## Results

### Comparisons of reference databases

The spatially normalized images of the 37 reference datasets used to build the normal databases for either software exhibited slight differences in FDG distribution, depending on the algorithm used for stereotactic normalization. As shown in Fig. 4a, differences between the mean image constructed with CAD4D’s B-spline spatial normalization algorithm (first row) and the mean image created with SPM’s normalization (second row) are not readily apparent. After calculation of the difference image (third row) however, a discrepancy in FDG distribution was found. We observed cortical differences of about 5–10 % between CAD4D- and SPM-normalized images. In CAD4D, the mean image and the cerebral cortex appear somewhat thickened as compared to SPM. The mean image created by SPM shows the peak FDG uptake in the cortices to be slightly shifted towards the margins of the brain as compared to CAD4D’s mean image. In addition, SPM’s normalization seems to enlarge the inter-hemispheric areas with high FDG uptake as compared to CAD4D normalization. As shown in Figs. 4b large differences in FDG distribution between CAD4D- and NEUROSTAT- normalized images were found mainly in subcortical areas (striatum, thalamus) and also in the range of 10–20 % in the cerebral cortex. The same effect was seen when comparing SPM- with NEUROSTAT-normalized images (see Fig. 4c).

### Statistical testing of non-Alzheimer patients’ data

Data of non-AD patients (group three) were analyzed using the reference databases. PET/CT images did not provide any evidence for hypometabolism. The statistical analyses conducted by CAD4D, SPM and NEUROSTAT as well found no relevant signs of AD-related hypometabolism (discriminating thresholds used: z-score >3 and cluster size >250 voxels, respectively). Therefore, this group was not subjected to further analysis.

### Statistical testing of Alzheimer patients’ data

Since discrepancies related to different spatial normalization procedures were observed in metabolic patterns of the reference databases, the impact of these procedures on analyzing single datasets from each of 20 patients with Alzheimer’s disease was investigated.

Hypometabolic brain regions as typical for AD were observed with all methods. The individual extent of hypometabolism was related to the developmental stage of the disease and differed slightly between the software packages used.

#### Comparison of the original results obtained with CAD4D and SPM in MNI space

The maximum z-score calculated by either method is shown in Table 1. In CAD4D, by default, the peak z-score was limited to 7.9. The z-scores were larger in CAD4D than in SPM, and the total numbers of hypometabolic voxels at threshold z-score >3.0 were on average 7 % larger in CAD4D(MNI) than in SPM(MNI).

#### Comparison of the original results of CAD4D, SPM and NEUROSTAT in Talairach space

The transformation of the CAD4D-database from MNI to Talairach space resulted in slightly lower z-scores. These were in the same range as with NEUROSTAT (in Talairach space). The transformation of the SPM-database into Talairach space led to maximal z-scores that were, on average, 28 % lower compared to NEUROSTAT and CAD4D(TAL) (see Table 1).

#### Comparison of directly calculated z-scores of SPM to CAD4D and NEUROSTAT

When comparing directly calculated z-scores from each AD patient and the SPM-normalized reference databases (“SPM calc” see Table 1) to those obtained with CAD4D in MNI and Talairach space, the z-scores were on average 12 % higher and the hypometabolic clusters at the threshold of z > 3.0 were significantly larger as compared to CAD4D (see Wilcoxon test statistics Table 1).

The maximal z-score per AD patient as calculated by CAD4D and SPM was located in the same brain area (temporo-parietal cortex) in 16 of 20 patients. With CAD4D, SPM and NEUROSTAT, this concordance was observed in 11 of 20 patients, since NEUROSTAT calculated the maximal hypometabolism to be located in the posterior cingulate cortex in five patients.

#### Categorical comparisons between reference databases and AD20 group

**Fig. 5:**
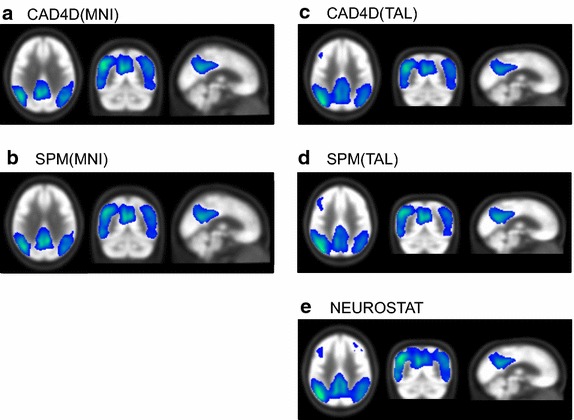
Results of SPM two sample t tests between the 37 reference datasets and 20 FDG-PET of AD patients. Images were spatially normalized images to the MNI space [**a** CAD4D(MNI), **b** SPM(MNI)], or to the Talairach space [**c** CAD4D(TAL), **d** SPM(TAL), **e** NEUROSTAT). For illustrative purposes, threshold t-maps (p < 0.001) were superimposed on FDG template. We used the same scaling for all t-maps shown (see also Table 2 for details)

We assessed the overall impact of the different spatial normalization procedure on testing the reference databases with a group of 20 AD patients, in order to detect disease-related hypometabolism. Table 2 summarizes the results of the five group comparisons (see Fig. 5a–e) between the 37 reference datasets and the AD20 group by using SPM’s two-sample t-test model and spatially normalized images of either CAD4D, SPM or NEUROSTAT.

**Table 2:** Evaluation of two sample t tests between 37 NC and 20 AD subjects

MNI	Cluster size (voxels)	Max. t-score	Voxel (x, y, z)	Brain area
CAD4D	24,350	13.04	(−40; −72; 42)	Left precuneus, parietal lobe
SPM	23,518	11.88	(−36; −74; 40)	Left precuneus, parietal lobe

TAL	Cluster size (voxels)	Max. t-score	Voxel (x, y, z)	Brain area
CAD4D(TAL)	13,552	11.93	(−38; −65; 27)	Left middle temporal gyrus, temporal lobe
SPM(TAL)	12,980	11.44	(−37; −73; 35)	Left precuneus, parietal lobe
NEUROSTAT	14,637	12.85	(−40; −71; 33)	Angular gyrus, temporal lobe

Extracted total hypometabolic cluster size at p < 0.001 (as displayed in Fig. 5), maximum t-score with position and corresponding brain area

Upper table: using CAD4D and SPM normalized images in MNI space

Lower table: using CAD4D, SPM and NEUROSTAT normalized images in Talairach space

In MNI space we found a common cluster of 20,042 voxels between CAD4D- (see Fig. 5a) and SPM-normalized images (see Fig. 5b), showing a cluster concordance of 88.2 % for SPM and 83.7 % for CAD4D, respectively.

In Talairach space the common cluster size between CAD4D(TAL), SPM(TAL) and NEUROSTAT (see Figs. 5c–e) consisted of 10,028 voxels. Concordance was detected for SPM(TAL) (overlap: 81.3 %) and CAD4D(TAL) (overlap: 77.9 %) as compared to NEUROSTAT (overlap: 72.0 %).

## Discussion

The present study investigated the effects of different spatial normalization and quantification methods on scanner-specific brain FDG-PET reference databases by comparing three different software packages.

### Constitution of the reference databases

The brain FDG-PET reference databases consisted of 37 healthy controls aged 50–85 years. We chose this age-matched database of elderly healthy controls since many patients developing neurodegenerative dementing disorders belong to this age group.

### Comparison of reference databases created with CAD4D, SPM or NEUROSTAT

The different spatial normalization procedures used by CAD4D and SPM (both operating in MNI space) resulted in different appearances of FDG-distribution in the resulting warped images. Compared to the Lobster algorithm implemented in CAD4D, the non-linear warping algorithm in SPM yielded a slightly reduced thickness of cortical structures, in line with the findings reported by Wenzel et al. [18]. This may artificially enlarge the cleft between the brain hemispheres and may lead to a slight “dislocation” of the striatum towards the brain outer surface in SPM.

After conversion of CAD4D- and SPM-generated databases into the Talairach space, the comparison of the three different stereotactic normalization methods revealed greater differences in FDG distribution appearance between NEUROSTAT and SPM and between NEUROSTAT and CAD4D than between SPM and CAD4D directly (s. Figure 4). This may be due to the similarity of SPM and CAD4D in the affine registration procedure. In contrast, NEUROSTAT uses a linear correction for the individual brain size compared to that of the standard dimensions of the Talairach atlas brain [28]. The conversion of the original FDG template from MNI to Talairach space as required when using CAD4D or SPM in the same space as NEUROSTAT may further affect the results of such a comparison.

Earlier studies compared different methods for analysis and spatial normalization of brain FDG-PET [17, 26, 31–33]. Hosaka and co-workers [17] reported a better concordance of voxels within the brain volume upon standardized MRI for SPM (88 %) compared to NEUROSTAT (85.3 %). Ishii et al. [26] found SPM to be more prone to atrophy, potentially leading to overrated ventricle sizes. Our present data appear to confirm the reports by the Ishii and Hosaka groups [17, 26]. Although the SPM option to use high resolution MRI for stereotactic normalization best reflected the hypometabolic pattern typical for AD compared to the PET-to-PET normalization [31]. However, the present study focuses on clinical situations in which additional MRI is not available. Notably, high-resolution MRI was conducted only in 28 % of our patients in 2013.

### Intensity scaling of FDG images

Scaling of intensity values within the images is a necessary step to remove differences due to inter-individual variations in the metabolic rate of brain glucose. The selection of an appropriate reference region for scaling has been widely discussed in the literature. As shown by some authors, scaling to an overall global mean (proportional scaling) may lead to artificial amplifications of the metabolism in non-affected regions [5, 34, 35] and thus to underestimation of hypometabolic areas in images of dementia patients [3]. Although proportional scaling is still in use [36], inter-subject scaling is often performed with respect to specific brain regions thought to be not or only slightly affected by neurodegenerative processes, e.g. the cerebellum [1, 3, 26] or sensorimotor cortical regions [3, 29]. Other methods include a data-driven approach (reference cluster method) as proposed by Yakushev et al. for group comparison [2]. While CAD4D scales the smoothed images to a common median intensity value derived from a predefined gray-white-matter mask [18], NEUROSTAT uses five different regions defined on the 2D projection images for scaling of the 3DSSP normal databases. Although proportional scaling may influence the performance of voxel-wise testing [2, 3, 34], we used the global scaled reference database in NEUROSTAT, since the correlation between the NEUROSTAT’s global scaling and CAD4D’s median was stronger (r = 0.967) than that of all other scaling options (e.g., NEUROSTAT cerebellum vs CAD4D median, r = 0.477).

### Smoothing of FDG images

In order to increase signal-to-noise ratio, all spatially normalized FDG images of healthy controls and patients were smoothed with a Gaussian kernel of 10 mm FWHM. According to our experience in brain FDG-PET imaging with different PET scanners, we chose a kernel with the size of about twice the scanner resolution. Testing with a smaller kernel size (8 mm) led to smaller hypometabolic clusters and a slightly lower z-score as compared to the same analysis using 10 mm smoothing (data not shown). In addition, spatial smoothing might influence the peak location within a hypometabolic cluster detected with SPM’s t-statistics [37].

### Statistical testing of Alzheimer subjects

When analyzing the results yielded by CAD4D, SPM or NEUROSTAT, substantial differences in peak z-score and extent of hypometabolic volume were found (see Table 1). Higher z-scores in CAD4D and NEUROSTAT as compared to SPM are due to the different z-score calculation method in SPM. Z-scores in CAD4D and NEUROSTAT were directly calculated, whereas z-scores in SPM were based on the calculated t-values and SPM’s t-to-z-transformation.

Since we used the “two sample t-test”-model in SPM for single subject analysis, a sample size larger than 30 and only one variance (of the reference group) contributing to this model, the t-values derived from single subject analysis should be close to their corresponding z-scores. Unexpectedly, the reported z-scores from t-transformation in SPM were markedly lower than their t-values. This is most probably due to the SPM function “spm_t2z.m”. Because of multiple comparison problems, SPM uses a different calculation of z for t-deviates with very small tail probabilities leading to underestimation of higher z-scores. Therefore, we additionally conducted a z-score calculation as used in CAD4D and NEUROSTAT (Table 1). Z-scores and cluster sizes obtained from direct z-score calculation of SPM-normalized images were similar or even larger than those found by CAD4D and NEUROSTAT. The smaller clusters and the lower z-scores obtained with the original SPM method may be addressed to the SPM model (single subject analysis) rather than to differences in spatial normalization methods.

As shown in Table 2, the group comparisons between the reference database and the AD 20 using spatially normalized images of CAD4D, SPM or NEUROSTAT, overall z-scores and cluster sizes of hypometabolism differed only slightly between the various methods.

Objective evaluation by direct calculation of z-scores of SPM warped images and CAD4D “regPET” images showed similar z-scores and cluster sizes, indicating that larger variations obtained by CAD4D and SPM may have resulted mainly from the different statistical methods. Here, very good agreement in peak detection of hypometabolism was found using CAD4D and SPM in 16 of 20 cases, localizing the maximal z-score in the parieto-temporal cortex. On the other hand, NEUROSTAT detected the highest z-score in 5 of these 16 subjects in the posterior cingulate cortex, probably due to the use of a different spatial normalization procedure as that used with SPM or CAD4D. In general, the direct calculations of z-scores appear notably sensitive for the detection of dementia-related hypometabolism.

### Study limitation

Due to the limited spatial resolution of PET (transaxial: ~4.5 mm full width of half-maximum (FHWM)), quantitative imaging is affected by the partial-volume-effect (PVE; i.e., the impact of surrounding structures on apparent tracer concentration in small regions). This effect may thus be important when imaging neurodegenerative disease patients who developed cortical atrophy, which was the case in some of our AD patients. We did not conduct PVE-correction (PVEc), since the respective software package developed by Quarantelli and co-workers [38] requires high-resolution MRI that commonly is not recorded in the clinical routine. However, Samuraki et al. [36] reported that FDG-uptake in brain areas, known to be affected by AD, e.g., the posterior cingulate cortex and the parieto-temporal lobes appeared to be reduced in these patients regardless of whether or not PVEc was applied. Furthermore, spatial normalization of brains with ventricular enlargement may introduce a potential misregistration of relative small brain nuclei, such as the caudate nucleus [39]. In addition, gray matter atrophy may influence the identification of the peak location and the extent of metabolic changes [26]. Although NEUROSTAT’s 3D-SSP calculations were developed to minimize atrophy effects [29], artificially high z-scores along the ventricular edges were still apparent in the medial 3D-SSPs. In CAD4D, an attempt was made to compensate for these artifacts by applying a predefined gray-white-matter mask.

## Conclusion

Based on scanner-specific and age-matched brain FDG-PET reference databases for CAD4D, SPM and NEUROSTAT, the single subject analysis of brain FDG-PET of each of 20 AD patients performed by either software showed similar patterns of hypometabolism in respective brain regions. The extent of hypometabolism and the location of its maximum z-score differed moderately even after changing the default settings in order to receive more comparability between the software packages. On the other hand, as a more objective evaluation, the directly calculated z-scores of SPM showed good concordance to those of CAD4D and NEUROSTAT. These results add an interesting point to the discussion on the comparability of brain FDG-PET studies: Even if the same subjects were used to create scanner-specific reference databases, the analysis of AD patient data appeared to be influenced by the calculation method rather than by the different spatial normalization methods used for creating a reference database with either of the software packages.
